# Luteolin-7-*O*-Glucuronide Improves Depression-like and Stress Coping Behaviors in Sleep Deprivation Stress Model by Activation of the BDNF Signaling

**DOI:** 10.3390/nu14163314

**Published:** 2022-08-12

**Authors:** Dajung Ryu, Hye-Jin Jee, Sang-Yoon Kim, Seung-Hwan Hwang, Gam-Bang Pil, Yi-Sook Jung

**Affiliations:** 1 College of Pharmacy, Ajou University, Suwon 16499, Korea; 2 Research Institute of Pharmaceutical Sciences and Technology, Ajou University, Suwon 16499, Korea; 3 KIURI Research Center, Ajou University School of Medicine, Ajou University, Suwon 16499, Korea; 4 R&D Center, Huons Co., Ltd., 55 Hanyangdaehak-ro, Ansan 15588, Korea

**Keywords:** luteolin-7-*O*-glucuronide, sleep deprivation, stress, depression, BDNF/TrkB/ERK/CREB signaling pathway

## Abstract

Stress exposure is a major risk factor for mental disorders such as depression. Because of the limitations of classical antidepressants such as side effects, low efficacy, and difficulty in long-term use, new natural medicines and bioactive molecules from plants with greater safety and efficacy have recently attracted attention. Luteolin-7-*O*-glucuronide (L7Gn), a bioactive molecule present in *Perilla frutescens*, is known to alleviate severe inflammatory responses and oxidative stress in macrophages. However, its antistress and antidepressant effects have not been elucidated. The present study aims to explore the antidepressant the effect of L7Gn on stress-induced behaviors and the underlying mechanism in a mouse sleep deprivation (SD) model. L7Gn treatment improved depression-like and stress coping behaviors induced by SD stress, as confirmed by the tail suspension test and forced swimming test. Furthermore, L7Gn treatment reduced the blood corticosterone and hippocampal proinflammatory cytokine levels which were increased by SD stress, and L7Gn also increased the mRNA and protein levels of hippocampal brain-derived neurotrophic factor (BDNF) which were reduced by SD stress. Additionally, treatment with L7Gn resulted in increases in the phosphorylation of tropomyosin-related kinase B (TrkB), extracellular signal-regulated kinase (ERK), and cAMP response element-binding protein (CREB), which are downstream molecules of BDNF signaling. These findings suggest that L7Gn have therapeutic potential for SD-induced stress, via activating the BDNF signaling.

## 1. Introduction

Numerous studies have shown that prolonged exposure to stress has detrimental effects on the central nervous system and leads to various mental disorders, such as learning and memory impairment, anxiety, and depression [1]. Depression is one of the most serious neuropsychiatric disorders induced by stress that reduces psychosocial functioning and quality of life [2,3]. It has been reported that people with depression experience 2.5 times more stress than normal individuals, and 80% of depression patients are preceded by stressful events [4]. Several studies have demonstrated a bidirectional association between depression and sleep disorders [5,6]. More than 90% of patients with depression suffer from sleep disturbances, which in turn can aggravate the severity of depressive symptoms [7].

Sleep is crucial for the maintenance of physiological functions, including metabolism, the endocrine system, cognition, and emotion [8]. Sleep deprivation (SD) is an important stress factor that negatively affects neurological as well as other physiological functions [9,10]. Studies using the murine SD stress model have demonstrated that SD causes temporary activation of the neuroendocrine stress systems and the hypothalamic-pituitary-adrenal (HPA) axis [11], leading to elevated levels of plasma glucocorticoids [12] and proinflammatory factors such as tumor necrosis factor (TNF)-α and interleukin (IL)-1β [13]. SD stress also contributes to depression-like behavior in relation to the disruption of synaptic plasticity [14]. Therefore, SD stress is considered to be a suitable model for inducing depression-like behavior. As a mechanistic molecule, brain-derived neurotrophic factor (BDNF) can modulate neurogenesis, reflecting the neuroplasticity hypothesis of stress-induced depression [15]. BDNF exerts antidepressant effects by binding to and activating tropomyosin-related kinase B (TrkB), a high-affinity protein kinase receptor [16,17,18].

**Figure 1:**
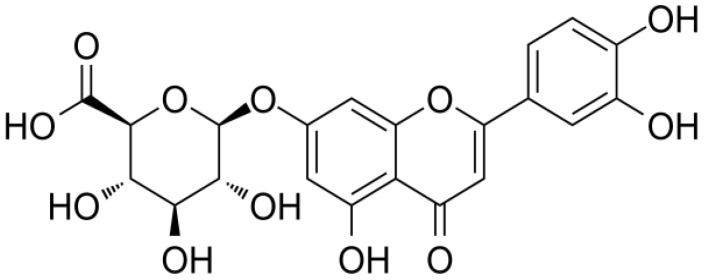
Chemical structure of Luteolin-7-*O*-glucuronide (L7Gn).

Nowadays, there is growing interest in the use of pharmacologically effective traditional plant extracts as antidepressants. The flavone glycoside luteolin-7-*O*-glucuronide (L7Gn, Figure 1) is one of the major compounds present in various plants, such as *Codariocalyx motorius*, *Ixeris dentata*, *Perilla frutescens*, and *Remirea maritima* [19,20,21,22]. L7Gn has been reported to have antimicrobial, antioxidant, antimutagenic, antigenotoxic, anti-inflammatory, and anti-arthritic activities [23,24,25]. A recent study reported that L7Gn can attenuate severe inflammatory responses and oxidative stress in macrophages [26,27]. However, the antistress and antidepressant effects of L7Gn have not been elucidated. The present study aims to examine whether L7Gn elicits antistress and antidepressant effects in an SD-induced mouse stress model; the underlying mechanism was further investigated.

## 2. Materials and Methods

### 2.1. Reagents

L7Gn was provided by Huons Co. Ltd. (Ansan, Korea). Fluoxetine (FXT) and N-[2-[(hexahydro-2-oxo-1H-azepin-3-yl)amino]carbonyl]phenyl-benzo[b]thiophene-2-carboxamide (ANA-12) were purchased from Sigma-Aldrich (Saint Louis, MO, USA). The plasma corticosterone ELISA kit was purchased from Enzo Life Sciences (Farmingdale, NY, USA). Anti-BDNF (#47808), anti-cAMP response element-binding protein (CREB) (#9104), anti-p-CREB (#9196), anti-extracellular signal-regulated kinase (ERK) (#9102), anti-p-ERK (#9101), anti-glyceraldehyde 3-phosphate dehydrogenase (GAPDH) (#2118), and anti-TrkB (#4603) antibodies were purchased from Cell Signaling Technology (Danvers, MA, USA). Anti-p-TrkB (ab229908) antibody was purchased from Abcam (Cambridge, MA, USA).

### 2.2. Isolation of L7Gn from Perilla frutescens Extarct

Dried leaves of *Perilla frutescens* var. (PF) were purchased from Daemyung pharm. Co., Ltd. (Seoul, Korea). The PF (500 g) was extracted twice with distilled water by refluxing at 95 ± 5 °C for 5 h. PF extract (PFE) was then evaporated under reduced pressure at 40 °C (Yield 28%). Subsequently, the PFE (100 g) was applied to an open glass column packed with Diaion HP-20 with H_2_O-MeOH in a gradient of 0–100% MeOH, thereby yielding 7 sub-fractions. Among them, fraction 7 (1 g) was chromatographed over sephadex LH-20 column chromatography with a 100% MeOH, and dried to obtain L7Gn (43.2 mg, 98.10%, purity). The L7Gn was identified by comparing ^1^H and ^13^C NMR spectra and previously reported data [28]. ^1^H-NMR (400 MHz, DMSO δc) δ 7.44 (^1^H, dd, *J* = 8.20, 2.30 Hz, H-6′), 7.43 (^1^H, d, *J* = 2.3 Hz, H-2′), 6.90 (^1^H, d, *J* = 8.20 Hz, H-5′), 6.79 (^1^H, *J* = 2.31 Hz, H-8), 6.74 (^1^H, s, H-3), 6.43 (^1^H, d, *J* = 2.10 Hz, H-6), 5.13 (^1^H, d, *J* = 7.27 Hz, H-1″), 3.80-3.10 (4H, m, H-2″, 3″, 4″ and 5″, overlap); ^13^C-NMR (100 MHz, DMSO, δc) δ 182.4 (C-4), 171.6 (C-6″), 165.7 (C-2), 161.6 (C-5), 163.4 (C-7), 157.4 (C-9), 150.5 (C-4′), 146.3 (C-3′), 121.7 (C-6′), 119.6 (C-1′), 116.5 (C-5′), 114.0 (C-2′), 105.8 (C-10), 103.6 (C-3), 100.1 (C-1″), 100.0 (C-6), 95.0 (C-8), 76.7 (C-3″), 73.4 (C-5″), 72.2 (C-2″), 70.8 (C-4″).

### 2.3. Animals

C57BL/6 male mice (7 weeks old, weighing 20–25 g) were purchased from Orient Bio Inc. (Seongnam, Korea). The animals were housed at 23 ± 1 °C and 60 ± 10% humidity with water and food ad libitum. The light and dark cycle of the room was altered every 12 h (lights on at 9:00 and off at 21:00). The animals were acclimatized for at least one week prior to the experiment. All experimental protocols were approved by the Institutional Animal Care and Use Committee (IACUC) of Ajou University (Approval Number 2020-0052).

### 2.4. SD Model

As described previously [29], SD was performed using a modified multiplatform method. The mice (*n* = 3–4) in each group were placed in water tanks (42 × 26 × 18 cm), containing eight cylindrical acrylic platforms (3 cm in diameter) each and filled with water until approximately 1 cm below the platform surface, for 72 h. The distance between the two columns was 5 cm, and the mice were able to move freely on each platform while jumping in the water-filled tank. During the 72 h SD period, the mice had free access to food and water. Mice in the control group were housed in normal cages.

**Figure 2:**
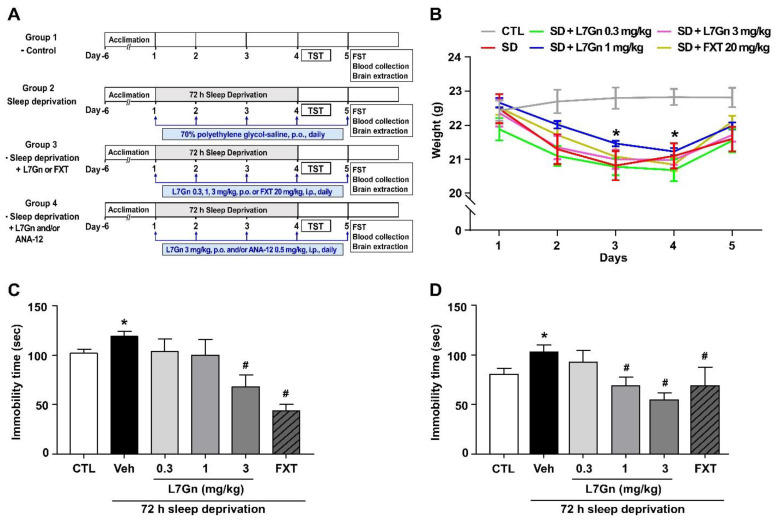
Effects of L7Gn on the behavior of sleep-deprived mice in TST (**B**) and FST (**C**). (**A**) Experimental design. L7Gn was dissolved in 70% PEG-saline, and FXT was dissolved in 0.9% physiological saline. Mice were administered vehicle (70% PEG), L7Gn (0.3, 1, and 3 mg/kg), or FXT (20 mg/kg) once daily for four–five days, followed by behavior tests. (**B**) Body weight: (two-way ANOVA: (Interaction: F (20, 150) = 1.258, *p* = 0.2165; Days: F (4, 150) = 15.18, *p* < 0.0001; Group: F (5, 150) = 16.64, *p* < 0.0001)) (**C**) TST: (one-way ANOVA: F (5, 38) = 7.627, *p* < 0.001) (**D**) FST: (one-way ANOVA: F (5, 35) = 3.76, *p* = 0.0079). Data are presented as mean ± SEM (*n* = 5–8/group). * *p* < 0.05, significantly different from the control group. # *p* < 0.05, significantly different from the vehicle group. CTL, control; Veh, vehicle; FXT, fluoxetine.

Figure 2A shows the experimental design. Except for the control group, all other groups were exposed to a 72 h SD using the multiple platform method. L7Gn was dissolved in 70% polyethylene glycol (PEG)-saline, and FXT was dissolved in 0.9% physiological saline. Mice were treated with vehicle (Veh, 70% PEG, p.o.), L7Gn (0.3, 1, and 3 mg/kg, p.o.), or FXT (20 mg/kg, i.p.) once daily for 5 days. The TrkB antagonist ANA-12 (0.5 mg/kg, i.p.) was dissolved in 1% dimethyl sulfoxide (DMSO) and administered 15 min before L7Gn treatment. Behavioral tests, i.e., tail suspension test (TST) and forced swimming test (FST), were performed between four and five days of vehicle, L7Gn, or FXT treatment. Vehicle or drugs were administered 30 min before all behavioral tests. The body weight of the mice in all groups was measured once daily for five days.

### 2.5. TST

The TST was performed to analyze depression-like behavior and the antidepressant -like effect of L7Gn. Briefly, mice were suspended, using sticky tape, approximately 1 cm from the tip of their tails, with their heads approximately 50 cm above the floor. The test was conducted for 6 min, during which mice remaining motionless were considered immobile. The duration of immobility was video recorded during the final 4 min; the first 2 min activity was considered ‘pre-test’.

### 2.6. FST

The FST is a widely used experimental protocol for evaluating stress coping behavior [30]. Each mouse was forced to swim in a 2 L beaker filled with water (25 ± 1 °C) at a depth of approximately 13 cm so that they could not touch the bottom or escape the beaker. Mice were individually placed in a beaker, forced to swim, and the total duration of floating was video recorded during a 6 min period. The mouse was judged to be immobile whenever it remained floating passively in the water. The duration of immobility was measured during the last 4 min of the test; the first 2 min were considered to be ‘pre-test’.

### 2.7. Plasma Corticosterone Level

After performing the behavioral tests, the mice were anesthetized by intraperitoneal administration of ketamine hydrochloride (100 mg/kg; Ketamine, Yuhan, Seoul, Korea) mixed with xylazine (10 mg/kg; Rompun, Bayer-Korea, Seoul, Korea), and blood was collected from the abdominal vein using a 1 mL syringe and added to 60 μL of 3.8% sodium citrate. Plasma samples were prepared by centrifugation of the collected blood samples (1000× *g* for 15 min) at 6 °C and then stored at −80 °C until experimentation. Plasma corticosterone levels were analyzed by Enzo Life Sciences ELISA kits (Farmingdale, NY, USA). Absorbance was measured using a Bio-Tek Synergy HT plate reader (Bio-Tek Instruments, VT, USA).

### 2.8. Western Blot Analysis

Hippocampal samples were homogenized and lysed in cold radioimmunoprecipitation assay buffer (150 mM NaCl, 1% NP-40, 0.25% Na-deoxycholate, 150 mM NaCl, 50 mM Tris-HCl, pH 7.4). The solution was centrifuged at 14,000 rpm and 4 °C for 15 min, and the concentration of proteins present in the supernatant was determined using a commercial BCA assay kit (Thermo Scientific, MA, USA) following the manufacturer’s instructions. Protein samples were loaded on an 8–12% SDS-polyacrylamide gel, electrophoresed, and then transferred onto PVDF membranes (Cytiva, Marlborough, MA, USA). After blocking with 5% skim milk, the membranes were incubated overnight at 4 °C with an appropriate primary antibody. The primary antibodies used were anti-BDNF (Cell Signaling, 1:1000), anti-p-TrkB (Abcam, 1:1000), anti-TrkB (Cell Signaling, 1:1000), anti-p-CREB (Cell Signaling, 1:1000), anti-CREB (Cell Signaling, 1:1000), anti-p-ERK (Cell Signaling, 1:1000), anti-ERK (Cell Signaling, 1:1000), and anti-GAPDH (Cell Signaling, 1:5000). PVDF membranes were washed with tris-buffered saline-Tween 20 and incubated with appropriate secondary antibodies for 1 h at room temperature. The band intensities were visualized using ECL detection reagents (Cytiva, Marlborough, MA, USA) and detected using an Amersham ImageQuant 800 system (Cytiva, Marlborough, MA, USA). GAPDH was used as the loading control. The ImageJ software was used for densitometric analysis of the western blot data (NIH, Bethesda, MD, USA).

### 2.9. Quantitative Reverse Transcription-PCR (qRT-PCR) 

RNA was isolated from the hippocampus of the mouse brain using TRIzol reagent (Invitrogen, CA, USA). The concentration of the extracted RNA was determined using a NanoDrop spectrometer (ND-LITE, Thermo Scientific, MA, USA), and the RNA samples were preserved at –80 °C until experimentation. One microgram of total RNA was reverse transcribed into cDNA using amfiRivert cDNA Synthesis Platinum Master Mix (GenDEPOT, CA, USA). The cDNA was used as a template for performing (qRT-PCR using amfiRivert qGreen Q-PCR Master Mix (GenDEPOT). The primers used were as follows: mouse TNF-α forward: 5′-CCTGTAGCCCACGTCGTAGC-3′, reverse: 5′-TTGACCTCAGCGCTGAGTTG-3′; mouse IL-1β forward: 5′-GCTTTCAGGGGAGGGCT-3′, reverse: 5′-GTGCTCTGGTTGCTCTCTGT-3′; mouse BDNF forward: 5′-TGGCTGACATTTTGAGCACG-3′, reverse: 5′-GCTCCAAAGGCATTGACTGC-3′; and rat GAPDH forward: 5′-CCATGGAGAAGGCTGGG-3′, reverse: 5′-CAAAGTTGTCATGGATGACC-3′. Gene expression was normalized to *GAPDH* mRNA levels.

### 2.10. Statistical Analysis

Data are presented as mean ± standard error of the mean (SEM). The body weight was analyzed using two-way ANOVA with Tukey’s post hoc test and other numerical data were compared using Student’s *t*-test or one-way ANOVA with Dunnett’s post hoc test for unpaired observations between the two groups. All statistical analyses were performed using Prism 7 for Windows (GraphPad Software Inc., La Jolla, CA, USA). For all analyses, statistical significance was set at *p* < 0.05.

## 3. Results

### 3.1. Effect of L7Gn on Depression-like and Stress Coping Behavior in the SD Stress Mouse Model

We evaluated the effect of L7G stress-induced behavior at various doses (0.3, 1, and 3 mg/kg, p.o.) in a murine model of SD-induced stress. From our preliminary experiments performed on L7Gn in various dose ranges from 0.1 to 10 mg/kg, we chose the doses of 0.3, 1, and 3 mg/kg, which exhibited dose-dependency. FXT, a selective serotonin reuptake inhibitor (SSRI) antidepressant, was used as the positive control. Our results showed that body weight was significantly decreased in SD mice, and this decreased body weight was not changed by L7Gn (Figure 2B). Sleep-deprived mice showed increased immobility time in the TST and FST compared to those in the control group, and this increased immobility time was significantly reduced upon treatment with 1 and 3 mg/kg L7Gn in the FST but only 3 mg/kg L7Gn in the TST (Figure 2C,D). These results indicate that L7Gn elicits antidepressant effect and increases active stress coping response to SD stress.

### 3.2. Effect of L7Gn Treatment on Plasma Corticosterone Level in Sleep-Deprived Mice

**Figure 3:**
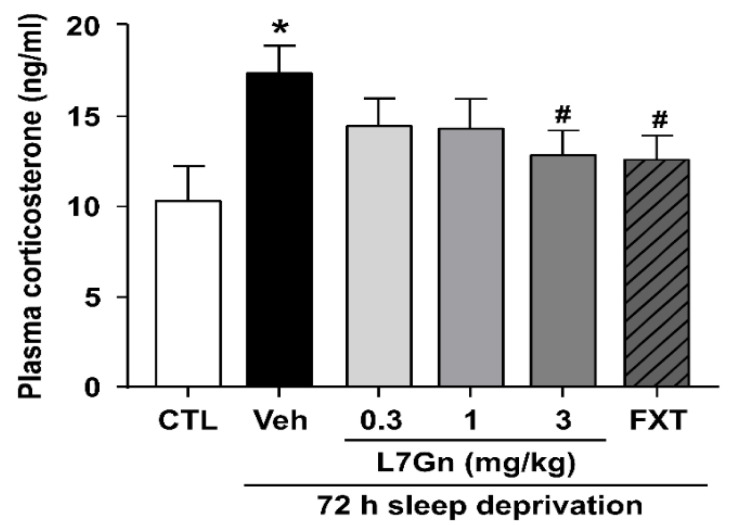
Effect of L7Gn (0.3, 1, and 3 mg/kg) on plasma corticosterone levels in sleep-deprived mice. L7Gn was dissolved in 70% PEG-saline, and FXT was dissolved in 0.9% physiological saline. Mice were administered vehicle (70% PEG), L7Gn (0.3, 1, and 3 mg/kg) or FXT (20 mg/kg) once daily for five days (one-way ANOVA: F (5, 25) = 2.247, *p* = 0.0809). Data are presented as mean ± SEM (*n* = 5–6/group). * *p* < 0.05, significantly different from the control group. # *p* < 0.05, significantly different from the vehicle group. CTL, control; Veh, vehicle; FXT, fluoxetine.

To examine the stress hormone-related response, we determined plasma corticosterone levels. Sleep-deprived mice showed a significant increase in plasma corticosterone levels compared with the control group (*p* < 0.05) (Figure 3). Treatment with L7Gn (3 mg/kg, p.o.) and FXT (20 mg/kg, i.p.) significantly suppressed the increase in plasma corticosterone levels (*p* < 0.05).

### 3.3. Effect of L7Gn on TNF-α and IL-1β Levels in Sleep-Deprived Mice

**Figure 4:**
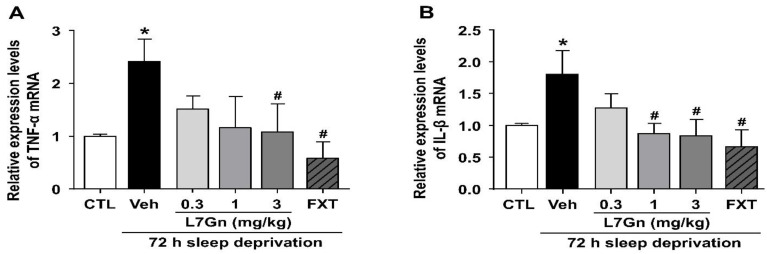
Effect of L7Gn on TNF-α and IL-1β mRNA levels in the hippocampus of sleep-deprived mice. L7Gn was dissolved in 70% PEG-saline, and FXT was dissolved in 0.9% physiological saline. Mice were administered vehicle (70% PEG), L7Gn (0.3, 1, and 3 mg/kg), or FXT (20 mg/kg) once daily for five days. The mRNA expression of (**A**) TNF-α and (**B**) IL-1β was assessed via qRT-PCR. (**A**) TNF-α: (one-way ANOVA: F (5, 26) = 2.649, *p* = 0.0459) (**B**) IL-1β: (one-way ANOVA: F (5, 29) = 2.789, *p* = 0.0356). Data are presented as mean ± SEM (*n* = 5–7/group). * *p* < 0.05, significantly different from the control group. # *p* < 0.05, significantly different from the vehicle group. CTL, control; Veh, vehicle; FXT, fluoxetine.

qRT-PCR was performed to investigate the effect of L7Gn on the production of the proinflammatory cytokines, TNF-α and IL-1β, in the hippocampus of sleep-deprived mice. The levels of TNF-α and IL-1β were significantly increased in sleep-deprived mice (*p* < 0.05), and these increased levels were significantly reduced by L7Gn (3 mg/kg, p.o.) and FXT (20 mg/kg, i.p.) treatment (Figure 4A,B).

### 3.4. Effect of L7Gn on BDNF mRNA Expression in the Hippocampus of Sleep-Deprived Mice

**Figure 5:**
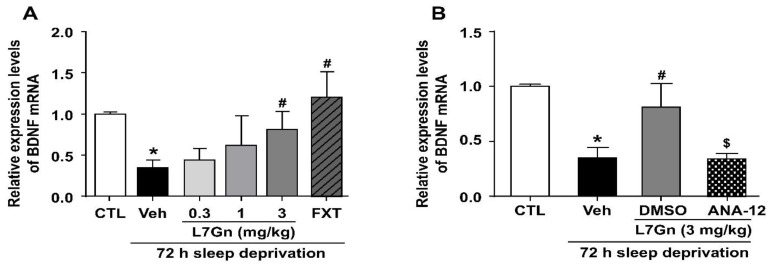
Effect of L7Gn on BDNF mRNA expression levels in the hippocampus of sleep-deprived mice. (**A**) L7Gn was dissolved in 70% PEG-saline, and FXT was dissolved in 0.9% physiological saline. Mice were administered vehicle (70% PEG), L7Gn (0.3, 1, and 3 mg/kg), or FXT (20 mg/kg) once daily for five days. (**A**) The mRNA expression of BDNF was assessed using qRT-PCR. (**B**) ANA-12 was dissolved in 1% DMSO. Mice were administered vehicle (70% PEG), L7Gn (3 mg/kg), or ANA-12 (0.5 mg/kg) once daily for five days. (**B**) The mRNA expression of BDNF was assessed via qRT-PCR. (**A**) BDNF: (one-way ANOVA: F (5, 14) = 2.734, *p* = 0.0631) (**B**) BDNF: (one-way ANOVA: F (3, 11) = 11.29, *p* = 0.0011). All data are presented as mean ± SEM (*n* = 3–4/group). * *p* < 0.05, significantly different from the control group. # *p* < 0.05, significantly different from the vehicle group. $ *p* < 0.05, significantly different from the L7Gn (3 mg/kg) group. CTL, control; Veh, vehicle; FXT, fluoxetine.

qRT-PCR was performed to evaluate the effect of L7Gn on BDNF mRNA expression in the hippocampus of sleep-deprived mice. BDNF mRNA levels were significantly reduced in the hippocampus of sleep-deprived mice (*p* < 0.05), and these levels were significantly increased upon L7Gn (3 mg/kg, p.o.) and FXT (20 mg/kg, i.p.) treatment (Figure 5A,B). Additionally, ANA-12 (0.5 mg/kg, i.p.) abolished the effects of L7Gn on BDNF mRNA levels.

### 3.5. Effect of L7Gn on the BDNF/TrkB/ERK/CREB Signaling Molecules in the Hippocampus of Sleep-Deprived Mice

**Figure 6:**
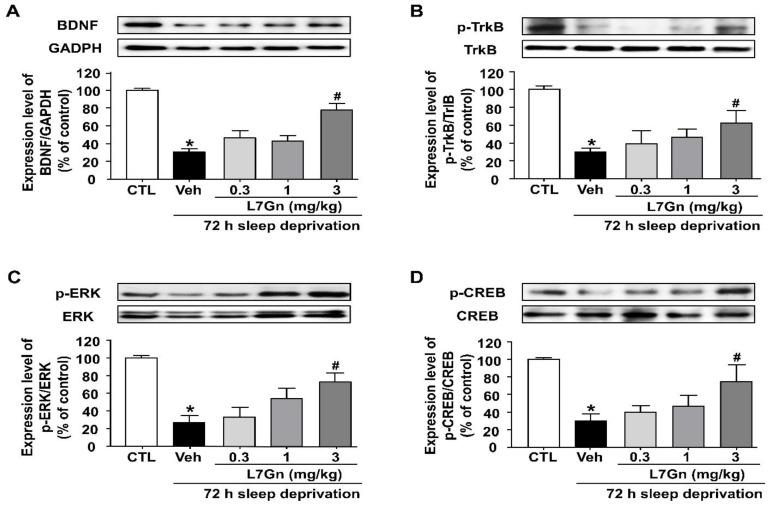
Effect of L7Gn on the BDNF expression and the phosphorylation of TrkB, ERK, and CREB in the hippocampus of sleep-deprived mice. L7Gn was dissolved in 70% PEG-saline. Mice were administered vehicle (70% PEG) or L7Gn (0.3, 1, and 3 mg/kg) once daily for five days. Western blotting and quantification of expression levels of (**A**) BDNF, (**B**) p-TrkB, (**C**) p-ERK, and (**D**) p-CREB. The expression level of BDNF was normalized against GAPDH and those of p-TrkB, p-ERK, and p-CREB were normalized against their unphosphorylated form. (**A**) BDNF: (one-way ANOVA: F (4, 15) = 24.33, *p* < 0.0001) (**B**) p-TrkB: (one-way ANOVA: F (4, 18) = 7.58, *p* = 0.0009) (**C**) p-ERK: (one-way ANOVA: F (4, 20) = 10.01, *p* = 0.0001) (**D**) p-CREB: (one-way ANOVA: F (4, 20) = 6.254, *p* = 0.0020). Data are presented as mean ± SEM (*n* = 4–5/group). * *p* < 0.05, significantly different from the control group. # *p* < 0.05, significantly different from the vehicle group. CTL, control; Veh, vehicle; FXT, fluoxetine.

To investigate the mechanism underlying the antistress effect of L7Gn, the effect of L7Gn on the expression of BDNF, p-TrkB, p-CREB, and p-ERK in the hippocampus was investigated. SD resulted in reduced BDNF, p-TrkB, p-CREB, and p-ERK expression levels, and these reduced levels were significantly increased upon L7Gn treatment (3 mg/kg, p.o.) (Figure 6).

### 3.6. Effect of ANA-12 on the Antidepressant Effect and Active Stress Coping Response Induced by L7Gn

**Figure 7:**
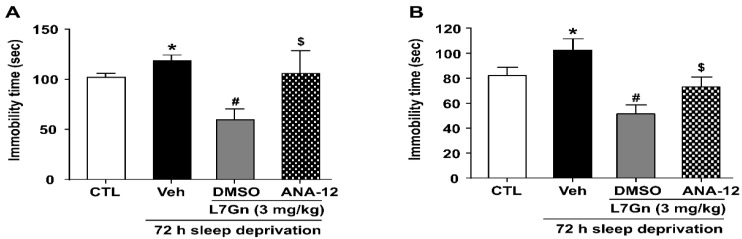
Effect of ANA-12 on the antidepressant effect and active stress coping response induced by L7Gn in sleep-deprived mice. L7Gn was dissolved in 70% PEG-saline, and ANA-12 was dissolved in 1% DMSO. Mice were administered vehicle (70% PEG), L7Gn (3 mg/kg), or ANA-12 (0.5 mg/kg) once daily for four–five days, followed by the (**A**) TST and (**B**) FST. (**A**) TST: (one-way ANOVA: F (3, 24) = 3.873, *p* = 0.0217) (**B**) FST: (one-way ANOVA: F (3, 22) = 8.152, *p* = 0.0008). Data are presented as mean ± SEM (*n* = 6–8/group). * *p* < 0.05, significantly different from the control group. # *p* < 0.05, significantly different from the vehicle group. $ *p* < 0.05, significantly different from the L7Gn (3 mg/kg) group. CTL, control; Veh, vehicle.

To assess the involvement of BDNF-TrkB in the mechanism of L7Gn’s effects, we evaluated the influence of ANA-12 (a TrkB antagonist) on the antidepressant effect and active stress coping response induced by L7Gn. L7Gn (3 mg/kg, p.o.) reduced the SD- induced elevation in immobility time in both the TST and FST compared to that in the vehicle group (*p* < 0.05) (Figure 7A,B). Theses effects of L7Gn (3 mg/kg, p.o.) were significantly inhibited by ANA-12 (0.5 mg/kg, i.p.) (*p* < 0.05).

### 3.7. Effect of ANA-12 Treatment on the L7Gn-Induced Upregulation of BDNF, p-TrkB, p-CREB and p-ERK

**Figure 8:**
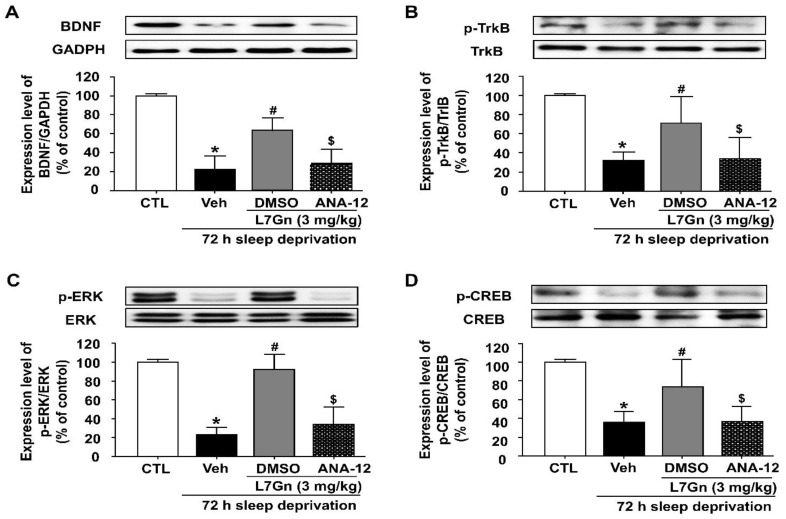
Effect of ANA-12 on the L7Gn-induced upregulation of BDNF, p-TrkB, p-CREB, and p-ERK in the hippocampus of sleep-deprived mice. L7Gn was dissolved in 70% PEG-saline, and ANA-12 was dissolved in 1% DMSO. Mice were administered vehicle (70% PEG), L7Gn (3 mg/kg) or ANA-12 (0.5 mg/kg) once daily for five days. Western blotting and quantification of expression levels of (**A**) BDNF, (**B**) p-TrkB, (**C**) p-ERK, and (**D**) p-CREB. The expression level of BDNF was normalized against GAPDH and those of p-TrkB, p-ERK, and p-CREB were normalized against their unphosphorylated form. (**A**) BDNF: (one-way ANOVA: F (3, 12) = 31.64, *p* < 0.0001) (**B**) p-TrkB: (one-way ANOVA: F (3, 12) = 12.3, *p* = 0.0006) (**C**) p-ERK: (one-way ANOVA: F (3, 11) = 10.97, *p* = 0.0012) (**D**) p-CREB: (one-way ANOVA: F (3, 12) = 12.11, *p* = 0.0006). Data are presented as mean ± SEM (*n* = 3–4/group). * *p* < 0.05, significantly different from the control group. # *p* < 0.05, significantly different from the vehicle group. $ *p* < 0.05, significantly different from the L7Gn (3 mg/kg) group. CTL, control; Veh, vehicle.

To assess the involvement of the BDNF/TrkB/ERK/CREB pathway in the antidepressant effect of L7Gn, we examined the effect of ANA-12 on L7Gn-induced upregulation of BDNF, p-TrkB, p-CREB, and p-ERK in the hippocampus of SD stressed mice. Treatment with L7Gn (3 mg/kg, p.o.) significantly increased BDNF, p-TrkB, p-CREB, and p-ERK protein levels, which were significantly reduced by ANA-12 (*p* < 0.05) (Figure 8).

## 4. Discussion

In the present study, we first found that L7Gn ameliorates depression-like and stress coping behavior in an SD stress mouse model, as shown by reduced immobility time in the TST and FST. Furthermore, L7Gn treatment reduced plasma corticosterone levels and the mRNA expression of TNF-α and IL-1β, which were elevated by SD stress. Additionally, L7Gn treatment increased the expression of BDNF, p-TrkB, p-CREB, and p-ERK, which were reduced by SD; all these effects of L7Gn were abolished by ANA-12, a potent TrkB antagonist. These results suggest that L7Gn exhibits an antidepressant effect and increases active stress coping response to SD stress, possibly by modulating the BDNF/TrkB/ERK/CREB signaling pathway.

SD is one of the stressors that can lead to depression by influencing physiological and psychological processes and synaptic plasticity, potentially leading to neurochemical changes in the brain [31]. It has been reported that SD stress induces abnormal activation of the HPA axis, leading to excessive corticosterone secretion and downregulation of hippocampal neurogenesis, ultimately leading to depression [12,32,33]. In the present study, we used a depression model induced by SD stress. Consistent with other studies, mice with SD-induced stress showed depression-like and stress coping behaviors, such as increased immobility time compared with normal mice in the TST and FST, which are typical behavioral tests that show animal depression-related patterns [34]. However, it has recently been proposed that FST behavior measures stress coping behavior without modeling depression in humans [30,35,36]. From this perspective, the behavioral changes measured by FST can be interpreted as reflecting stress coping adaptability. SD mice also exhibited increases in the levels of plasma corticosterone and brain tissue proinflammatory cytokines. Since classic antistress and antidepressant drugs have many limitations such as side effects, low efficacy, and difficulty for long-term use, new natural medicines or bioactive molecules from natural plants that are safe and effective have recently been attracting attention [37]. Here, we explored the antidepressant effects of L7Gn, a bioactive molecule isolated from *Perilla frutescens*, in an in vivo SD-induced depression model. We demonstrated that L7Gn-treated mice showed a significant decrease in immobility time in the FST and TST, suggesting an ameliorating effect of L7Gn on depression-like and stress coping behaviors. Additionally, plasma corticosterone levels were significantly lower in L7Gn-treated SD mice than in vehicle-treated SD mice. In a stressful situation, the corticotropin-releasing hormone (CRH), which is synthesized from the hypothalamus, activates the HPA axis. Upon activation, CRH is released into the anterior pituitary where it stimulates the release of adrenocorticotropic hormone (ACTH) leading to the secretion of corticosterone from the adrenal cortex [38]. Therefore, further study is needed to examine whether L7Gn can affect the HPA axis to elucidate the correlation between antidepressant effect of L7Gn and its effect on the HPA axis.

SD stress has also been reported to induce depression by promoting the expression of proinflammatory cytokine genes, such as IL-1β and TNF-α, which are well-known stress markers [13]. TNF-α and IL-1β are associated with neuronal plasticity, which is further linked with the cognitive abilities of patients with depression and brain innate immunity [39,40]. Additionally, many studies have reported that a reduction in BDNF levels is a significant factor contributing to the occurrence of depression [41]. BDNF, a neurotrophin, is an essential component of the signaling molecule responsible for neuronal survival, synapse formation, and synaptic plasticity in the brain [16,42]. Therefore, BDNF has a potential role in the treatment of neuropsychiatric disorders, including intellectual disability, schizophrenia, autism, and mood disorders [43]. Various stress events result in reduced BDNF levels in the hippocampus, whereas chronic treatment with conventional antidepressants increases BDNF levels. Moreover, the binding of BDNF to the TrkB receptor is known to promote key downstream signaling pathways, mainly those leading to phosphorylation and activation of ERK and CREB [44,45]. A reduced activity of the BDNF downstream signaling pathway induces susceptibility to depression in rodents, whereas activation of this pathway causes antidepressant-like effects in animal models of depression [46,47]. Previous studies have shown ERK expression is significantly decreased in the prefrontal cortex of depressed and suicidal patients [48], and that antidepressants can ameliorate depression-like behavior by elevation of p-ERK [49]. Other studies have further shown a remarkable decrease in the expression level of CREB in the hippocampus of patients with depression and chronically stressed mice [50,51]. Taken together, these findings indicate that the BDNF/TrkB/ERK/CREB signaling pathway may be a potential therapeutic target for depression.

In the present study, we found that the reduced BDNF mRNA and protein expression levels in the hippocampus of SD-induced depressive mice were significantly increased upon L7Gn treatment. Additionally, L7Gn treatment increased the expression level of BDNF, p-TrkB, p-ERK, and p-CREB, which were reduced by SD (Figure 5, Figure 6 and Figure 8). Furthermore, the antidepressant effects of L7Gn (3 mg/kg) in TST and FST were significantly abolished by ANA-12 (Figure 7). ANA-12 treatment further inhibited the effects of L7Gn (3 mg/kg) by affecting the expression of BDNF, p-TrkB, p-ERK, and p-CREB in the mouse hippocampus (Figure 5B and Figure 8). These results suggest that L7Gn can upregulate the BDNF/TrkB/ERK/CREB signaling modules in the hippocampus of SD-induced depressive mice.

There are several reports that luteolin is detected in the blood and brain tissues after the peripheral injection, suggesting the ability of luteolin to pass through the BBB [52,53]. In fact, the luteolin glucuronide, such as L7Gn, has been reported to be deconjugated to luteolin by β-glucuronidase in an in vivo inflammatory mice and rat model [54,55]. Given that the SD stress is known to induce inflammation by activating various inflammatory factors, it is possible to hypothesize that L7Gn can cross the BBB in the form of deconjugated luteolin in our SD model, but further study is needed to clarify this hypothesis.

## 5. Conclusions

In conclusion, our study shows that L7Gn exerts an antidepressant effect and in-creases active stress coping response to SD stress in mice, and the underlying mechanism may involve, at least partially, activation of the BDNF signaling. Although further studies are needed to further elucidate the association of L7Gn with the HPA axis, this study provides new insights into the therapeutic potential in SD-induced stress.

## Data Availability

All generated data are available within this manuscript.
